# A Novel Approach to Improve the Estimation of a Diet Adherence Considering Seasonality and Short Term Variability – The NU-AGE Mediterranean Diet Experience

**DOI:** 10.3389/fphys.2019.00149

**Published:** 2019-03-05

**Authors:** Enrico Giampieri, Rita Ostan, Giulia Guidarelli, Stefano Salvioli, Agnes A. M. Berendsen, Anna Brzozowska, Barbara Pietruszka, Amy Jennings, Nathalie Meunier, Elodie Caumon, Susan Fairweather-Tait, Ewa Sicinska, Edith J. M. Feskens, Lisette C. P. G. M. de Groot, Claudio Franceschi, Aurelia Santoro

**Affiliations:** ^1^Department of Experimental, Diagnostic and Specialty Medicine, University of Bologna, Bologna, Italy; ^2^Interdepartmental Centre “L. Galvani”, University of Bologna, Bologna, Italy; ^3^Division of Human Nutrition, Wageningen University & Research, Wageningen, Netherlands; ^4^Faculty of Human Nutrition and Consumer Sciences, Warsaw University of Life Sciences-SGGW, Warsaw, Poland; ^5^Norwich Medical School, University of East Anglia, Norwich, United Kingdom; ^6^Centre Hospitalier Universitaire de Clermont-Ferrand, Clermont-Ferrand, France; ^7^Institute of Neurological Sciences (IRCCS), Bologna, Italy

**Keywords:** diet assessment, Bayesian statistics, hierarchical models, Mediterranean-like diet, seasonality, regression to the mean, inflammaging

## Abstract

In this work we present a novel statistical approach to improve the assessment of the adherence to a 1-year nutritional intervention within the framework of the NU-AGE project. This was measured with a single adherence score based on 7-days food records, under limitations on the number of observations per subject and time frame of intervention. The results of the NU-AGE dietary intervention were summarized by variations of the NU-AGE index as described in the NU-AGE protocol. Food and nutrient intake of all participants was assessed by means of 7-days food records at recruitment and after 10 to 14 months of intervention (depending on the subject availability). Sixteen food groups and supplementations covering the dietary goals of the NU-AGE diet have been used to estimate the NU-AGE index before and after the intervention. The 7-days food record is a reliable tool to register food intakes, however, as with other tools used to assess lifestyle dietary compliance, it is affected by uncertainty in this estimation due to the possibility that the observed week is not fully representative of the entire intervention period. Also, due to logistic limitations, the effects of seasonality can never be completely removed. These variabilities, if not accounted for in the index estimation, will reduce the statistical power of the analyses. In this work we discuss a method to assess these uncertainties and thus improve the resulting NU-AGE index. The proposed method is based on Hierarchical Bayesian Models. This model explicitly includes country-specific averages of the NU-AGE index, index variation induced by the dietary intervention, and country based seasonality. This information is used to evaluate the NU-AGE index uncertainty and thus to estimate the “real” NU-AGE index for each subject, both before and after the intervention. These corrections reduce the possibility of misinterpreting measurement variability as real information, improving the power of the statistical tests that are performed with the resulting index. The results suggest that this method is able to reduce the short term and seasonal variability of the measured index in the context of multicenter dietary intervention trials. Using this method to estimate seasonality and variability would allow one to obtain better measurements from the subjects of a study, and be able to simplify the scheduling of diet assessments.

**Clinical Trial Registration:** www.ClinicalTrials.gov, identifier NCT01754012.

## Introduction

Aging is characterized by a peculiar low-grade chronic inflammatory status, not caused by the presence of pathogens or allergens. This condition was termed inflammaging (Franceschi et al., 2000) and is due to the increasing load of exogenous and endogenous stressors eliciting unabated inflammatory responses which are shown to substantially contribute to the pathogenesis of many, if not all, age-associated diseases and to the progression of the aging process (Franceschi and Campisi, 2014; Kennedy et al., 2014).

A coherent set of epidemiological data shows that the Mediterranean diet (MedDiet), the cultural dietary model typical of the populations living in the Mediterranean region, has beneficial effects in the prevention of a variety of age-related diseases (Korre et al., 2014) in which low-grade, chronic inflammation/inflammaging plays a major role. The MedDiet could represent a powerful tool to profoundly re-modulate the systemic inflammatory balance on a long-term scale by slowing down the age-related increase in the production of inflammatory molecules and by favoring adaptive anti-inflammatory responses (Trichopoulou et al., 2003; Trichopoulou, 2004; Sofi et al., 2008; Martucci et al., 2017; Ostan et al., 2018).

This is the conceptual framework of the European Commission-funded project NU-AGE (“New dietary strategies addressing the specific needs of the elderly population for healthy aging in Europe”; grant agreement 266486, NU-AGE Consortium. European Project NU-AGE^1^, Accessed March 14, 2018 (Santoro et al., 2014) aimed to test the hypothesis that inflammaging can be counteracted or slowed down by a 1 year complete dietary intervention, based on a Mediterranean dietary pattern newly designed to meet the nutritional needs of different elderly populations in Europe (NU-AGE diet) (Berendsen et al., 2014). In this context, 1240 volunteers, aged 65–79 years, were enrolled in 5 European countries (Italy, France, Poland, the Netherlands, and the United Kingdom). At the beginning and end of the 1 year NU-AGE dietary intervention, all subjects underwent a comprehensive evaluation, and dietary intake was assessed by means of 7-days food records completed by the participants (Berendsen et al., 2014; Ostan et al., 2018). The NU-AGE index that is used to describe the adherence to the prescribed diet is described in Berendsen et al. (2018).

The 7-days food record is a reliable tool to evaluate and monitor short-term current dietary intake (Ostan et al., 2018) and to guide participants of dietary intervention studies toward changing their intakes, but, as with other assessment methods, it is affected by intra- and inter-individual fluctuations due to several factors including the personal motivation of the subjects, their health status, and seasonality. Intra-individual fluctuations are simply due to the natural variability in one’s diet, so a measurement over two consecutive weeks could lead to significantly different estimates of the NU-AGE index (or any equivalent summary index). These intra-individual fluctuations are a source of uncertainty in the analysis of the effect of a dietary intervention, and need to be estimated and accounted for in the analyses. Given the usage of this index as a numerical value in the analyses done in NU-AGE, the estimation of this uncertainty need to be done in a quantitative way. Calibration methods such as 24-h recalls and a biomarkers could be helpful in assessing biases in the 7-days food records, but using them to quantitatively estimate the uncertainty in a derived index would require an even more complex statistical model.

The estimation of this uncertainty is central in the statistical modeling, as different sources of variability can have profound effects on the results of the analyses. In particular, in a repeated measurement with the goal of estimating the effect of a dietary intervention (or any intervention in general), one has to include the effect of the “regression to the mean” in the analysis. If not accounted for, regression to the mean can severely distort the analysis (Barnett et al., 2005). This “regression to the mean” happens when in a time series analysis the intrinsic variability of the measurement process is not explicitly accounted for in the analysis and is therefore confused with the real signal. Regression to the mean would present itself as a negative correlation between the baseline value and the observed variation, and an increase in the variability of the effect of the diet in each individual.

The consequence of this is a reduction in the power of the statistical tests that can be performed and, consequently, the certainty of the knowledge that can be derived from the study. This typically means that, when this uncertainty is not properly accounted for, nutritional studies need to include a great number of subjects to make up for the additional uncertainty, and the effect size appears to be smaller than what it actually is.

The second effect that could potentially distort the analyses is the seasonality effect. This is due to different nutritional patterns during the year. This seasonal variation, if not accounted for, could lead to estimating a distorted effect of the dietary intervention in the score, thus reducing the validity of the assumptions. This problem is usually avoided by designing the study such that the baseline (T0) and after intervention (T1) 7-days food records are 1 year apart, so that this effect would cancel out. There are three limitations with this design: the need for coordination among different countries, need for coordination inside a single recruitment center between the different subjects, and enforcing the 1 year gap with all of the individual subjects. These requisites are difficult to satisfy entirely, and therefore the seasonality will become a new source of variability in the data. Given that the subjects all had two recording phases each, it is not possible to estimate an individual effect of the seasonality, but it is still possible to evaluate a country-wise effect and remove it from each individual measurement.

The aim of this paper is to evaluate how the intra- and inter- individual variability could potentially distort the results of analyses based on 7-days food records, along with an estimate of the magnitude of the seasonality effect in NU-AGE index, and to propose a model to correct these issues. The proposed method is based on Hierarchical Bayesian Models, a Bayesian equivalent of the multilevel mixed effect model, informed by literature driven knowledge about the plausible effects of such a dietary intervention. This model explicitly includes the country specific baseline values, NU-AGE index, and country based seasonality. This information is used to evaluate the measurement variability and thus the “real” subject-specific NU-AGE index at baseline and the “real” effect of the NU-AGE dietary intervention on the NU-AGE index after 1-year. Based on this statistical method, an experimental approach to nutritional studies using 7-days food records is discussed.

## Materials and Methods

### NU-AGE Study Design

This study was performed using baseline and 1 year follow up data of the NU-AGE dietary intervention study. The NU-AGE study was carried out in five European study centers (Bologna in Italy, Norwich in the United Kingdom, Wageningen in the Netherlands, Warsaw in Poland, and Clermont-Ferrand in France). Recruitment started in April 2012 and finished in January 2014 including 1,296 healthy European men and women aged 65–80 years. Subjects have been recruited continuously over the 12 months of the year, and the final visit was performed 1 year (10–14 months, according to the subjects’ availability) after the recruitment. The rationale and design of this intervention study are described in detail elsewhere (Berendsen et al., 2014; Santoro et al., 2014). Participants completed 7-days food records to provide information about their dietary intake. The study protocol was approved by local medical ethics committees at all study sites and the NU-AGE study is registered with clinicaltrials.gov since December 21st 2012, NCT01754012. Local ethical approval was provided by the Independent Ethics Committee of the Sant’Orsola-Malpighi Hospital Bologna (Italy), the National Research Ethics Committee – East of England (United Kingdom), the Wageningen University Medical Ethics Committee (Netherlands), the Bioethics Committee of the Polish National Food and Nutrition Institute (Poland) and South-East 6 Person Protection Committee (France). All study procedures were in accordance with the ethical standards of the Helsinki Declaration. All participants gave written informed consent before participating.

### Dietary Assessment by 7-Days Food Records

Dietary intake was estimated by means of 7-days food records completed by the participants according to a protocol designed for healthy elderly participants of the NU-AGE project (Berendsen et al., 2014; Ostan et al., 2018). Briefly, participants received instructions before starting to fill in the 7-days food records by a trained interviewer. NU-AGE food records were provided in a structured format, with tables for each day and eating occasion. The amounts of each food could be measured with a kitchen weighing scale, using household measures, or photographs. Participants were recommended not to change eating habits during the week of registration. At the end of the recording period, the 7-days food records were checked by a trained dietician/research nutritionist. Consumed foods were coded according to standardized coding procedures and translated into nutrients (such as sodium and vitamins, see Ostan et al., 2018) by the use of software exploiting local food composition tables (NEVO 2011 in The Netherlands, WISP in The United Kingdom, INRAN and IEO in Italy, NFNI in Poland and CIQUAL French food composition table in France).

### Dietary Intervention

Participants randomized into the NU-AGE diet group received monthly individual dietary counseling from a trained dietician/research nutritionist aiming to meet the NU-AGE Food Based Dietary Guidelines (FBDG’s, Berendsen et al., 2014, 2018). Dietary counseling was based on the Motivational Interview technique and the Stages of Changes model (Prochaska and Diclemente, 1982; Miller and Rollnick, 2002). To sustain adherence to the NU-AGE FBDG’s, participants randomized into the NU-AGE diet group received commercially available foods meeting the NU-AGE dietary guidelines. Additionally, participants in the NU-AGE diet group received vitamin D supplementation (10 μg/day). Participants in the control group received a leaflet with national dietary guidelines that is generally available in Italy^2^, the United Kingdom^3^, the Netherlands^4^, Poland^5^, and France^6^. The details of NU-AGE dietary intervention are described elsewhere (Berendsen et al., 2014, 2018).

### NU-AGE Index

The NU-AGE index is meant to reflect the adherence to the NU-AGE diet on the basis of the 16 recommendations jointly integrated into NU-AGE FBDG’s provided to participants randomized into the NU-AGE diet group. The NU-AGE index comprises recommendations of minimum amounts to consume for fruits, vegetables, legumes, low-fat dairy, low-fat cheese, fish, low-fat meat and poultry, nuts, olive oil, fluids, and vitamin D supplementation. Two recommendations give a definition of minimum and maximum intake frequencies for whole grains and eggs and three recommendations refer to components to limit (alcohol, salt and sweets) (Berendsen et al., 2018).

Specific NU-AGE food groups were created based on the underlying principles of the NU-AGE diet (i.e., low-fat, low-sodium, high MUFA and PUFA, high fibers), using country-specific food grouping systems. All individually consumed foods were grouped into NU-AGE food groups in each country, resulting in comparable food groups across countries.

A continuous scoring system was created (Berendsen et al., 2018) to assign participants a score according to their level of adherence to each of the 16 NU-AGE diet components. Cut-off values for each NU-AGE diet component were based on the NU-AGE FBDG’s. For whole grains, fruits, vegetables, legumes, low-fat dairy, low-fat cheese, fish, low-fat meat and poultry, nuts, olive oil and fluids, a score ranging from 0 to 10 could be obtained, where higher scores indicate greater intakes of a specific food component. For diet components to limit (alcohol, sodium and sweets), participants with lower intakes received 10 points and participants with higher intakes received 0 points. The maximum level of intake for several NU-AGE diet components was established based on the country-specific population’s intake distribution (i.e., 85th pct. for sodium and sweets, 100th pct. for whole grains and low-fat meat). In total, the NU-AGE index was obtained by adding these 16 scores, and ranged from 0 to 160 points, ranking participants according to their adherence to the NU-AGE diet.

### Study Population

Participants who had not completed the 7-days food records at baseline, those with missing data on supplement use, incomplete diet data, and those with an energy intake below 500 kcal (*n* = 0) or exceeding 3500 kcal (*n* = 1) (Rhee et al., 2015) were excluded. A total of 1133 participants (241 from Italy, 251 from the United Kingdom, 235 from the Netherlands, 222 from Poland, and 184 from France) were considered for the analysis (Berendsen et al., 2018).

### Data Analysis

#### Estimating Distortion of NU-AGE Index Estimation Due to Intrinsic Fluctuations

To eliminate the effect of the measurement variability in the estimation of the NU-AGE index, the analysis needs to explicitly incorporate all of the sources of variance in the observed values. In this analysis the variance of the NU-AGE index of each subject is assumed to be the sum of four sources of variability: measurement variability, country variability, seasonality, and inter-personal variability. The analysis explained in this work attempts to describe each source of variability, and to include it in the model, to assess the real effect of the NU-AGE dietary intervention, removing spurious effects from other sources of variability.

The method relies on Bayesian statistics and multi-layer hierarchical methods to estimate these effects. In Bayesian statistics one assumes that the measurement (in this case the observed 7-days food record) is a known quantity, and describes other quantities as only partially known (the subject intrinsic adherence to the NU-AGE diet, represented by the NU-AGE index), where the amount of information is described by a probability distribution of the more plausible values. The observed quantities are used to update these beliefs on the uncertain values based on the measurements available. In this approach, every measurement of the 7-days food record provides more information on the underlying value of the adherence to the diet.

Before discussing the complete analysis, we performed a sanity check on the results we expect to have from a simplified model of the measurement process. We used a computer simulation of an idealized situation to assess what the expected results should be. In this model we considered only the measurement variability and the interpersonal variability, as these are suspected to be the greatest contributors to the overall variance. Varying the relationship between these two sources of variability, we get a curve that predicts the negative correlation between the baseline value and the effect of the intervention that would be found in an idealized study similar to NU-AGE.

The model used to estimate this effect is the following: one can assume a population with the same size as the NU-AGE one, with baseline values distributed as a normal distribution with mean 0 and standard deviation 1. One then introduces a random variation between T0 and T1 distributed in the same way. On top of these two values, there is an experimental uncertainty modeled as a normal distribution with mean 0 and a standard deviation λ, the free parameter of this model. The uncertainty is added to both the measurement at T0 and T1, with two independent realizations. One can then simulate the expected correlation between the variation between T0 and T1 with the observed value at T0 depending on the value of λ. This result can be inverted to estimate the value of λ given the observed negative correlation.

#### Estimation of the Seasonality Component of the NU-AGE Index

The study was designed so that the dietary assessments would be at 12 months of distance, to reduce the effect seasonality on the data. This is a common approach to the problem, but still suffers from two limitations: (1) the sampling will have some months of variation due to the human component and (2) the inter-subject variability will be artificially increased due to difference in the period of the year when each subject is collected.

The simplest model for the seasonality is a sinusoidal oscillation with a yearly period, where the peak intensity and the peak location during the year can vary. Shorter periods (such as 6 months) were not used as they were considered of little practical value while adding non-trivial complexity to the model. This oscillation modifies the average value of the NU-AGE index during the year. The exact parametrization used in the model is discussed in the Supplementary Materials, Section S1.

The seasonality is estimated as an effect at the level of countries, not individuals, as the effect is small and there is not enough data to estimate it reliably. It is also reasonable to expect that most of the effects that influence the seasonality, such as climate and availability of food, are shared among the population.

#### Estimating Similarities of Subjects Within Countries

To estimate the similarities between different individuals and across the countries involved in the study, we employed hierarchical Bayesian models. Hierarchical models allow one to describe the similarities in a group of individuals, and inform the observed values of each one of them based on this similarity. This means that groups of subjects (in this case based on nationality) sharing similar values are brought closer together by the method. This is especially useful in studies where there are few observations for each subject (and thus high uncertainty in the real value), as these methods provide additional power to the analysis (Gelman et al., 2004). This method corrects mostly data points for which the data is scarce, while leaving subjects with more information (and thus more power) almost unchanged.

The hierarchy comes from the description of the plausible values that each individual could have (the prior distribution) as dependent from the plausible values that are observed across the population. This model can be repeated for higher hierarchies: in this study we used one level to describe the subjects based on their population, and one level to describe all the populations based on the overall results.

These models were implemented using the procedures described in Betancourt et al. (Betancourt and Girolami, 2013), using the NUTS algorithm implemented by the Pymc3 library (Hoffman and Gelman, 2014; Salvatier et al., 2016). More details about the statistical model are included in the Supplementary Materials, Section S2. The software used in the analysis is described in the Supplementary Materials, Section S3.

#### Model Description

The observed data for the model is the adherence to the NU-AGE diet (NU-AGE index) estimated by the 7-days food records as described in the previous section. Data was collected at recruitment and after 1 year of dietary intervention or habitual diet, for the control group). These two time points are referred to as T0 and T1. The exact date of the sampling was converted from day of the year to a fraction of the length of the year, to be used for the estimation of the seasonality. The specific distributions and parameters used for all of the parameters of the models are described in the Supplementary Materials, Section S4. The NU-AGE index of the subjects before the study is considered constant until T0 and is referred to as the baseline value of the index. This represents the value around which the measured NU-AGE index would fluctuate due to the normal variability of the diet of each individual.

The basic assumption for the estimation of the effect of the intervention is that the control group had a negligible variation in their baseline score compared with the NU-AGE diet group (subjects that followed the NU-AGE diet). This was estimated as roughly 5% of the effect due to participating in the study as a subject. This hypothesis is fundamental to allow one to estimate the variability in the measurement of the baseline, due to the 7-days food records method.

The effect of the intervention on the NU-AGE index is estimated with a two layer hierarchical model. There is a first general layer of NU-AGE index common to the whole European population, then a level specific for each individual country and then down to the level of each individual subject. This baseline value is used both for T0 and T1, modified by the seasonality effect and the adherence to the NU-AGE diet (for T1).

The baseline NU-AGE index was estimated using a single layer hierarchical model where each country has its own center distribution, that informs about the adherence to NU-AGE at the individual level. The single layer hierarchical level was used instead of a two level one (including a common effect for all countries) as the big difference that the French samples had compared with the others made the convergence of the model problematic without introducing additional terms in the model, assessing the probability of outlier behavior.

The seasonality effect is described as detailed in the previous section as the sum of two oscillating components, and the regression determines the weights of these two components. The base value is shared among the various populations, and then each individual one is determined using a hierarchical model.

## Results

### Regression to the Mean Null Model

Regression to the mean would present itself as a negative correlation between the baseline value and the observed variation, and an increase in the variability of the effect of the NU-AGE diet in each individual. Both effects are present in the dataset:

**Table:** 

	**Mean difference**	**Difference std**	**Diff. - T0 *r*-value**
**Control group**	2.0	15.2	-0.41
**NU-AGE diet group**	23.3	16.9	-0.49


**FIGURE 1:**
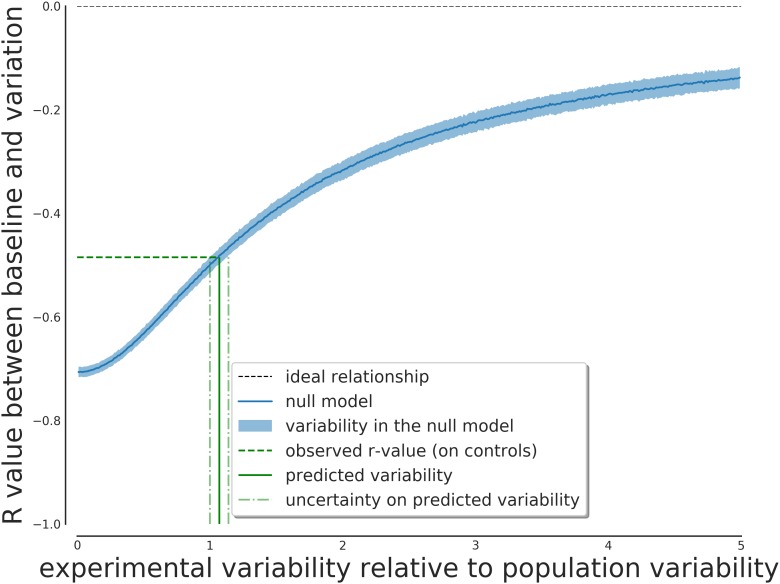
Expected effect on the score correlation depending on the ratio between within-variability and between-variability of the subjects. This relationship can be inverted to obtain the expected ratio given the observed correlation value.

The results of this simulation are shown in Figure 1. As it can be seen from Figure 1, the expected correlation between the T0 and T1 measurements depends strongly on the ratio between the within-individual and between-individual, ranging from -0.7 and converging to 0 when the within-variability increases.

One can use the observed negative correlation as input for this model to obtain the predicted value of the ratio of the two uncertainties. Given the negative correlation of -0.49, this ratio is predicted to be greater than one (1.07), so a greater measurement variability compared to interpersonal variability is expected.

The predicted value of λ from the model was 1.07, with an IQR of [1.01, 1.14]. The result from the complete model has a best value of 1.03, with an IQR of [1.01, 1.08] (with a 95% interval of [0.95, 1.15]). The results of the null model is thus compatible with the results from the complete Bayesian model, giving strong support to the regression to the mean hypothesis and the need for the correction.

**FIGURE 2:**
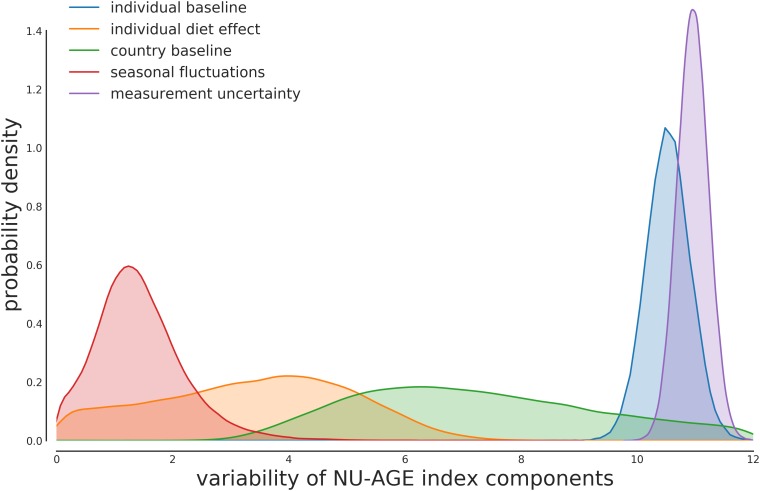
Posterior distribution for the variability components of each part of the model. The majority of the variability is composed of the individual variability in the baseline value and the measurement uncertainty. The measurement uncertainty is slightly higher than the between-individual variability, as expected from the model described in Figure 1.

The predicted within-individual to between-individual variability is thus consistent with the estimates that can be predicted from the null model of regression to the average. The estimated within-individual variability is compatible with previous estimates in elderly populations such as (Fukumoto et al., 2013) of approximately 20% of the base value (Figure 2).

### Country-Wise Average NU-AGE Index

**FIGURE 3:**
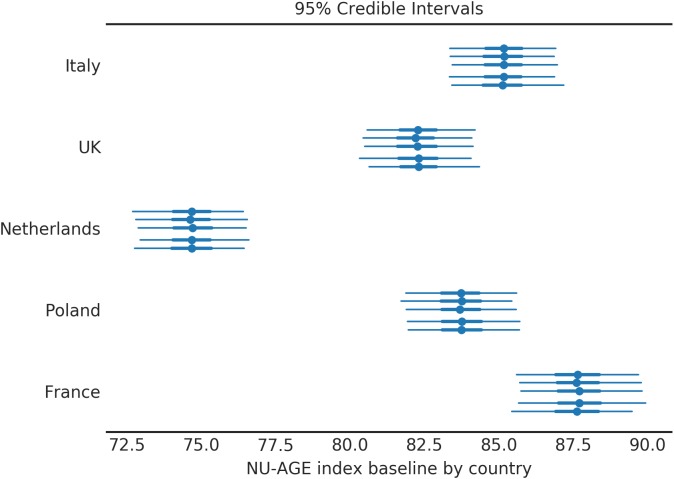
Baseline NU-AGE index for the population by country. Each country is divided in 5 segments to assess the variability among simulations (each simulation corresponds to one line). The central point represents the mean of the posterior distribution, the thick line represents the 50% HDI, the thin line represents the 95% HDI.

The proposed model uses country-wise averages to inform the estimation of the individual subjects’ adherence to the NU-AGE diet. These values represent the average NU-AGE index that can be expected from a person in that country (Figure 3).

For each country the average values of NU-AGE index at T0 are indicated; the simulation was repeated 5 times (as discussed in the Supplementary Materials, Section S4), and the results are shown in Figure 3. Four countries out of five have a compatible value of baseline (T0) NU-AGE index, with the exception of the Netherlands.

**FIGURE 4:**
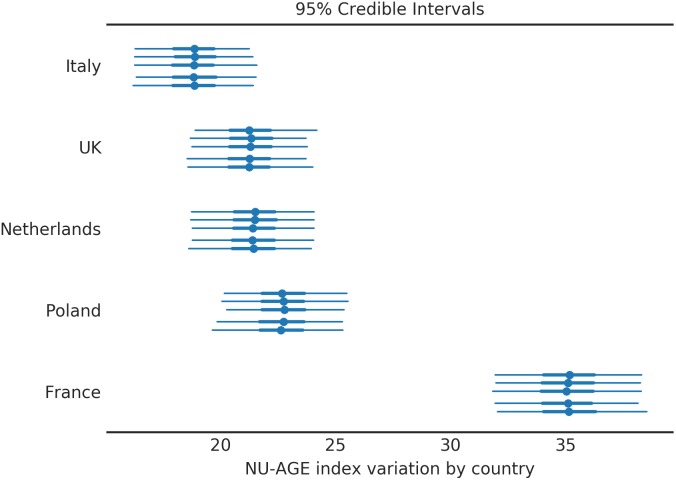
NU-AGE index variation after 1 year intervention for the population divided by country. Four countries out of five behave similarly, while the French subjects had a noticeably higher NU-AGE index. Each country is divided into 5 segments to assess the variability among simulations (each simulation corresponds to one line). The central point represents the mean of the posterior distribution, the thick line the 50% HDI, the thin line the 95% HDI.

The variation of NU-AGE index after the intervention is shown in Figure 4. Four of the five countries have similar effects from the intervention of 20 points (equivalent to 12.5% of the maximum amount, or 25% of the baseline average (Figure 3). The only exception is the subjects in France, where the effect is almost double that of other countries. This similarity of the effect of the NU-AGE diet across different countries suggests that the intervention itself was successful and the measure reliable. The significant difference between France and the other countries is a confirmation that this is a real effect and not due simply to a shrinkage induced by the analysis method.

### Corrected Values of NU-AGE Index

The central result of the model is an estimate of the baseline value of the NU-AGE index of each participant, both for T0 and T1. This estimate removes the effect of the seasonality and reduces the influence of the uncertainty of the estimation method.

**FIGURE 5:**
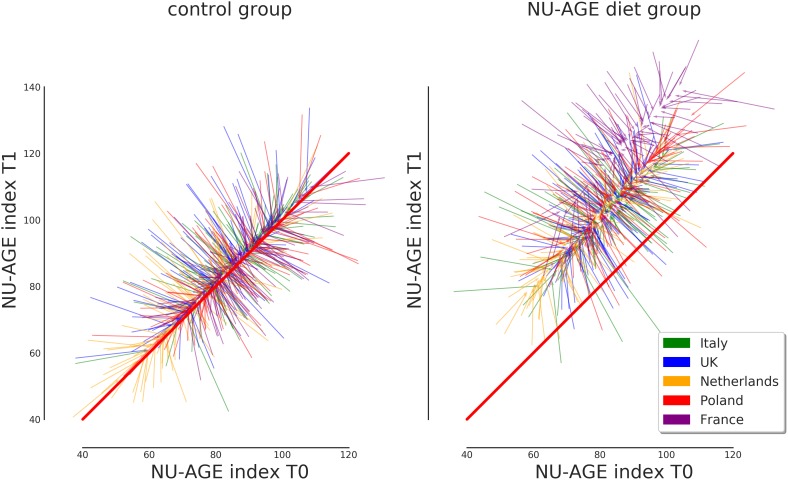
Representation of the effect of the correction on the NU-AGE index at T0 and T1 for both control and NU-AGE diet intervention group. Each arrow represents a subject, starting from the non-corrected values at (T0, T1) and arriving at the post-correction values. Longer arrows represent a stronger correction, while the barely visible arrows at the center of each cloud represent subjects whose NU-AGE index has not been corrected. The diagonal red line represents identical NU-AGE index before and after the intervention study.

The relationship between the original values and the corrected ones for T0 and T1 is shown in Figure 5 for the control and the NU-AGE diet group, respectively. The graph represents the effect of the correction on the NU-AGE index values for each subject. It can be seen that for the controls the values converge toward the central trend line of identity between T0 and T1 (as it was assumed for the controls). The NU-AGE diet group, represented on the right side of Figure 5, can be seen converging toward a diagonal line parallel to the identity one, shifted by an amount equal to the one reported in Figure 4. The corrected values lie close to the country line, with a reduction of the extreme values to a more average one: this is a consequence of the uncertainty in the measurement of the NU-AGE index combined with the population average: extreme values are more plausibly just random fluctuations rather than real deviance from the population average.

### Seasonality Estimation

**FIGURE 6:**
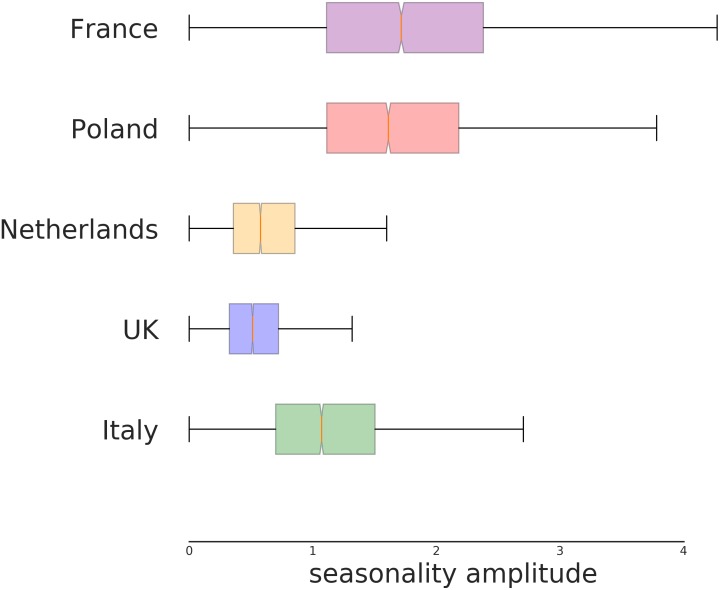
Seasonal amplitude of the variation of NU-AGE index according to country. This represents the amplitude of maximum variation (above and below the yearly average) for the seasonality effect, independently from the estimated period of higher compliance. The difference among the countries is significant. In particular, the NU-AGE index in both England and the Netherlands seems to have a lower seasonal variability compared to the other countries.

The effect of the seasonality, estimated for each country, is shown in Figure 6, 7. Figure 6 indicates the intensity of the effect as amplitude of the oscillation. The difference of NU-AGE index between the extremes of the seasonality is about 4 points on the national average for some countries (France and Poland). Considering that the differences among countries are in the order of magnitude of 6 to 8 points (as can be seen in Figure 2), this effect can be considered relevant in the estimation of the adherence to the NU-AGE diet and, therefore, should be included in the model.

**FIGURE 7:**
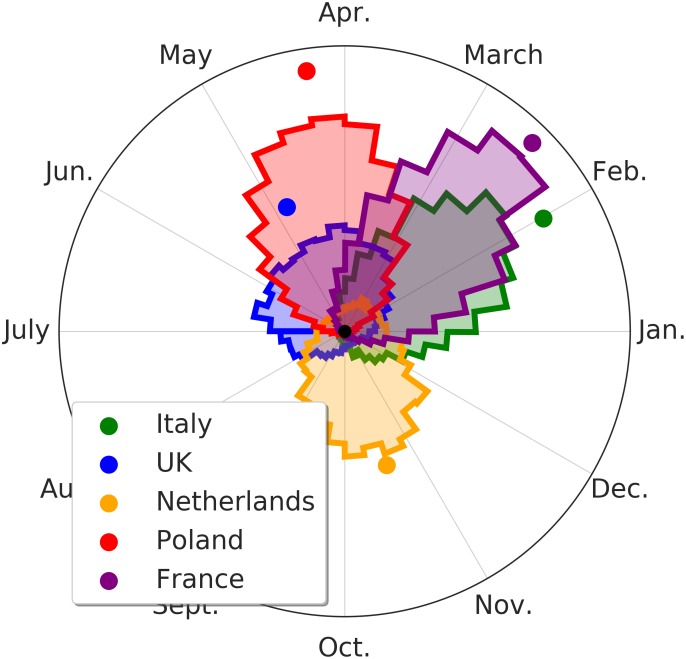
Month of maximum variation of the seasonality of the NU-AGE index. The colored area represents the distribution of plausible values of the NU-AGE index for the peak period. Higher and thinner peaks represent a higher degree of certainty on the estimation of the peak period. The colored dots represent the average peak location (and degree of certainty). The NU-AGE index in the English and the Netherlands subjects undergoes a lower seasonality effect.

In Figure 7 the specific period of NU-AGE index peak are shown. As it can be seen, the countries with the lower effect are also the ones with the less clear peak.

It is worth pointing out that the estimated seasonality is country-wise and not at individual level. This is because each subject performed the 7-days food records at T1 at 1 year of distance from T0 (within a month or two of variability). This means that the effect of the seasonality cannot be estimated for each individual (as it would require more temporal points) but only on average among participants in the same country. This probably hides the individual variability in seasonality effects, reducing the magnitude of the overall country-wise effect.

## Discussion

Methods for assessing diet composition and quantifying nutrient intakes are essential components of any study aimed to detect significant associations between diet, health outcomes and risk of disease, as well as the effect of a dietary intervention trial. This work shows that the processing phase of data from 7-days food records requires careful handling. The main issues that arise from this analysis are: the necessity of a control design to assess the variability of the quantity that one aims to measure (here: NU-AGE index) and an estimation of the seasonality effects in the diet (measured as NU-AGE index).

The results of the analyses show that both the seasonality and the 7-days food record short term fluctuations can be reliably measured in the NU-AGE dataset. In particular the short term fluctuations in the measurements of a single individual have a similar magnitude as the variability across individuals in the same country. This means that, without accounting for these fluctuations during the analysis, the effective variance on the data is double what it really is to estimate the population-wide effect. This means that the statistical power of any analysis done without the correction is equivalent to a study with half the number of subjects that properly address the intra-individual fluctuations.

This is an important consideration when a new clinical study is designed to assess the effect of a dietary intervention, both using 7-days food records or any other kind of measurement of the diet. The measurement variability assessment could be performed using both a control group or a repeated measure for the same subjects before (or after) the start of a dietary intervention. The possibility of estimating seasonality components in the diet adherence score (both for the overall score and the individual components of the scores) means that repeated measures do not need to be necessarily spaced 1 year apart. On the opposite, multiple measurements at shorter times could improve the quality of estimation of both seasonality and uncertainty, improving therefore the possibility of identifying the real effect of the dietary intervention. These corrections reduce the possibility of misinterpreting measurement variability as real information, improving the power of the statistical test that could be performed with the resulting dietary index.

The results of this study have some limitations from a cohort perspective and from a statistical one. The population enrolled in the NU-AGE study is composed of volunteers, making them a non-representative sample of the general population, as they were on average more interested in health and nutritional topics, and better educated. In particular this can be seen in the France population, where the baseline adherence and the improvement with the intervention were significantly higher than from all the other European centers. From a statistical perspective, the model has two major underlying assumptions: (1) the greatest sources of variability were the ones included in the model and not some other, unaccounted ones; (2) the likelihood of the index can be represented with a Normal distribution. This is reasonable in the case of the NU-AGE index, as it was generated by the sum of 16 different items (with a 0–10 range) and this justifies the assumption by virtue of normal distribution convergence.

The results of this work might be employed in the design of future similar studies. The following two considerations can be extrapolated from these results. First, to assess individual level variations, given the variability in the measurements, it would be better to assess the index at least 2 times for each time point (before and after the study period). Second, intermediate measurements of diet adherence, while useful to assess the behavior of the subjects of the study, should not be used to assess the adherence variation as performed in the NU-AGE study, as including them would need a more complicated model, voiding any improvement that could be gained from them. This would also improve the estimation of the seasonality, allowing also for an individual level assessment, instead of just an average for the whole country. Being able to estimate the effect of seasonality on the 7-days food records would also allow a less restrictive design of the study. This would allow the researchers to better accommodate the needs of the subjects of the study and, at the same time, reduce the necessity to design the study to have evenly spaced data collection moments to completely remove the effect of seasonality from the data analysis. The improvement on the statistical power from the reduction in variability would also mean that less subjects would be required to obtain the same level of accuracy and significance levels.

To summarize, the benefits of including such a statistical approach to estimate seasonality and measurement variability would allow researchers to obtain more accurate statistics from the same number of subjects in a study and not be forced to assess diet at 1 year intervals.

## Author Contributions

CF conceived the NU-AGE project. AS coordinated the NU-AGE data collection across centers. AAMB and LdG designed the dietary intervention. AAMB, RO, BP, AB, and EC followed the dietary intervention. AB was responsible for the dietary intake data validation. AAMB, RO, BP, AB, AJ, NM, EF, SF-T, and ES were responsible for data collection and analysis. EG, RO, GG, AS, and SS worked on the statistical analysis and its validation. EG drafted and finalized the manuscript. All authors were accountable for all aspects of the work in ensuring that questions related to the accuracy or integrity of any part of the work were appropriately investigated and resolved, critically revised the manuscript for important intellectual content, and agreed on the final draft of the manuscript.

## Conflict of Interest Statement

The authors declare that the research was conducted in the absence of any commercial or financial relationships that could be construed as a potential conflict of interest.

## Footnotes

1 http://www.nu-age.eu

2 IEA-Council for agricultural research and economics 2003 [cited 2012 03-08-2011]. Italian Dietary Guidelines]. Available from: http://nut.entecra.it/

3 Department of Health. Report on Health and Social Subjects 41 Dietary Reference Values for Food Energy and Nutrients for the United Kingdom; Health Council of the Netherlands: London, United Kingdom, 1991.

4 Health council of the Netherlands. Guidelines for a healthy diet 2006. The Hague: 2006.

5 Jarosz M, Bulhak-Jachymczyk B. Normy zywienia czlowieka. Podstawy prewencji otylosci i chorob niezakaznych / Nutrition recommendations. Basis for the prevention of obesity and non-communicable diseases. Warsaw: Wydawnictwo Lekarskie PZWL / National Food and Nutrition Institute 2008.

6 Ministère du Travail dlEedlS. French National Nutrition and Health Program 2011–2015 2011.
