# Supplementary material for: A Novel Approach to Improve the Estimation of a Diet Adherence Considering Seasonality and Short Term Variability – The NU-AGE Mediterranean Diet Experience
Source: Front Physiol. 2019 Mar 5;10:149. doi: 10.3389/fphys.2019.00149 (PMC6413567; doi:10.3389/fphys.2019.00149)
Supplement: Supplementary file 1 [file Data_Sheet_1.doc]

Supplementary Materials

# S1 - Seasonality model

The seasonality P has a natural description in terms of two parameters: the intensity A of the variation and the phase $\phi$ of the variation (when the peak is):

$$P=A cos(\omega t+\phi)$$

where $\omega$ has the value and measurement unit required for the cosine to have a one-year period, depending on the unit of measurement of time. This formulation has bad convergence properties for the analysis, and is better reparametrized using the cosine summation formula as:

$$P=C cos(\omega t)+S sin(\omega t)$$

where, to recover the original formulation, one can use the following expressions:

$$A=\sqrt{C^{2}+S^{2}}$$

$$\phi=atan_{2}(S,C)$$

# S2 - Non central hierarchical model implementation

As shown by Betancourt et al. (Betancourt and Girolami, 2013), from a computational perspective hierarchical models require a specific reparametrization to avoid convergence issues when integrated using Hamiltonian Montecarlo methods such as the one used in this work (using the default integration algorithm of the pymc3 library (Hoffman and Gelman, 2014; Salvatier et al., 2016).

This parametrization uses 3 parameters for each hierarchical layer: one parameter that represents the position of the grand mean of the groups, modeled in most cases with a normal distribution with given mean and standard deviation; one parameter that models the dispersion between the groups, modeled by a half normal distribution with mean 0 and standard deviation of 10 (weakly informative prior); one parameter that models the offset between the different groups, modeled as a normal distribution with mean 0 and standard deviation 1. The value of each group is then defined as the grand mean plus the product of the spread times the group offset.

This modelization allows for the inter group spread to collapse to 0 (representing a condition where the data are not informative enough to discriminate between different groups) without collapsing the offset, avoiding strong variations in the geometry of the parameter space and thus avoiding convergence issues.

# S3 - Software used

All models were implemented in Python 3.6 (Python Software Foundation, <https://www.python.org/>) using IPython (Pérez and Granger, 2007) and Jupyter (Kluyver et al., 2016). Data were processed using the pandas (McKinney, 2010), sympy (Meurer et al., 2017), scipy (Jones et al.) and numpy (Travis E, 2006) libraries. The model was implemented in PyMC3 (Salvatier et al., 2016) and the results were visualized using matplotlib (Hunter, 2007) and seaborn (Waskom et al., 2017)

# S4 - Statistical model

## S4.1 - Stochastic variables used in the model

Seasonality parameters:

- SeasonSigma = HalfNormal(sd=5)
- SeasonSinCenter = Normal(mu=0, sd=10)
- SeasonSinOffset = Normal(mu=0, sd=1) [one per country]
- SeasonCosCenter = Normal(mu=0, sd=10)
- SeasonCosOffset = Normal(mu=0, sd=1) [one per country]
- SeasonCosWeight = SeasonCosCenter + SeasonSigma * SeasonCosOffset
- SeasonSinWeight = SeasonSinCenter + SeasonSigma * SeasonSinOffset
- SeasonT0 = SeasonCosWeight * cos(timeT0) + SeasonSinWeight * sin(timeT0)
- SeasonT1 = SeasonCosWeight * cos(timeT1) + SeasonSinWeight * sin(timeT1)

Baseline value of the NU-AGE index

- CountryCompliance = Normal(mu=80, sd=40) [one per country]
- IndividualCenter = [one per individual] equal to the one of the country
- IndividualSigma = HalfNormal(sd=40)
- IndividualOffset = Normal(mu=0, sd=1) [one per individual]
- IndividualComplianceT0 = IndividualCenter + IndividualSigma * IndividualOffset

Effect of the dietary intervention on the diet group:

- CountryDiffCenter = Normal(mu=0, sd=40)
- countryDiffSigma = HalfNormal(sd=10)
- countryDiffOffset = Normal(mu=0, sd=1) [one per country]
- CountryDiff = CountryDiffCenter + countryDiffSigma * countryDiffOffset
- StatusWeight = 1 for subjects, 0.05 for controls
- IndividualDiffCenter = [one per individual] StatusWeight * country weight,
- IndividualDiffSigma = HalfCauchy(beta=40)
- IndividualDiffOffset = Normal(mu=0, sd=StatusWeight) [one per individual]
- IndividualDiff = IndividualDiffCenter + IndividualDiffSigma * IndividualDiffOffset
- IndividualComplianceT1 = IndividualComplianceT0 + IndividualDiff

Observations:

- complianceObsStd = HalfNormal(sd=40)
- measureT0 = Normal(mu= individualcomplianceT0 + seasonT0, sd=complianceObsStd)
- measureT1 = Normal(mu= individualcomplianceT1 + seasonT1, sd=complianceObsStd)

the model parameters were estimated using a NUTS Montecarlo with acceptance rate of 0.95, 4000 steps of tuning and 5000 draws. The model was run 5 times to assess convergence and the final results were evaluated combining the results of all the 5 simulations.

## S4.2 - Effective sample size statistics

|  | max | median | min |
| --- | --- | --- | --- |
| CountryCompliance | 56589 | 52578 | 39263 |
| CountryDiff | 904 | 709 | 378 |
| CountryDiffCenter | 897 | 897 | 897 |
| IndividualDiff | 85000 | 85000 | 889 |
| IndividualDiffOffset | 85000 | 85000 | 85000 |
| IndividualDiffSigma | 581 | 581 | 581 |
| IndividualOffset | 85000 | 85000 | 85000 |
| IndividualSigma | 159 | 159 | 159 |
| SeasonCos | 77177 | 51055 | 30315 |
| SeasonCosCenter | 48841 | 48841 | 48841 |
| SeasonCosOffset | 82853 | 73822 | 57019 |
| SeasonSigma | 18376 | 18376 | 18376 |
| SeasonSin | 65477 | 51972 | 40632 |
| SeasonSinCenter | 52404 | 52404 | 52404 |
| SeasonSinOffset | 78525 | 76664 | 75240 |
| SeasonT0 | 78271 | 52173 | 29928 |
| SeasonT1 | 78337 | 52320 | 29933 |
| complianceObsStd | 114 | 114 | 114 |
| countryDiffOffset | 50414 | 12337 | 6726 |
| countryDiffSigma | 2784 | 2784 | 2784 |
| individualComplianceT0 | 85000 | 85000 | 85000 |
| individualComplianceT1 | 85000 | 85000 | 85000 |

## S4.3 - Gelman Rubin R statistics

|  | max | median | min |
| --- | --- | --- | --- |
| CountryCompliance | 1.000067 | 1.000015 | 0.999974 |
| CountryDiff | 1.000009 | 0.999987 | 0.999979 |
| CountryDiffCenter | 1.000308 | 1.000308 | 1.000308 |
| IndividualDiff | 1.000440 | 0.999992 | 0.999971 |
| IndividualDiffOffset | 1.000195 | 0.999986 | 0.999971 |
| IndividualDiffSigma | 1.001174 | 1.001174 | 1.001174 |
| IndividualOffset | 1.000045 | 0.999984 | 0.999971 |
| IndividualSigma | 1.000078 | 1.000078 | 1.000078 |
| SeasonCos | 1.000067 | 1.000009 | 0.999990 |
| SeasonCosCenter | 1.000012 | 1.000012 | 1.000012 |
| SeasonCosOffset | 1.000043 | 1.000018 | 0.999983 |
| SeasonSigma | 1.000111 | 1.000111 | 1.000111 |
| SeasonSin | 1.000093 | 1.000030 | 0.999987 |
| SeasonSinCenter | 1.000049 | 1.000049 | 1.000049 |
| SeasonSinOffset | 1.000053 | 1.000004 | 0.999990 |
| SeasonT0 | 1.000095 | 1.000027 | 0.999977 |
| SeasonT1 | 1.000095 | 1.000026 | 0.999977 |
| complianceObsStd | 1.000110 | 1.000110 | 1.000110 |
| countryDiffOffset | 1.000179 | 1.000119 | 1.000061 |
| countryDiffSigma | 1.000006 | 1.000006 | 1.000006 |
| individualComplianceT0 | 1.000058 | 0.999984 | 0.999971 |
| individualComplianceT1 | 1.000118 | 0.999986 | 0.999971 |

all values are below the commonly accepted threshold of $1.01$.
